# Tranexamic acid use and the risk of postoperative delirium after spine surgery: a retrospective cohort study

**DOI:** 10.1186/s12871-026-04302-z

**Published:** 2026-10-05

**Authors:** Paula Petersein, Juliane Schulze, Johannes Ehler, Antje Vogelgesang, Johanna Ruhnau, Robert Fleischmann

**Affiliations:** 1 Department of Neurology, University Medicine Greifswald, Ferdinand-Sauerbruch-Straße, 17475 Greifswald, Germany; 2 Department of Anesthesiology and Intensive Care Medicine, University Hospital Jena, Jena, Germany

**Keywords:** Tranexamic acid (TXA), Postoperative delirium (POD), Neuroinflammation, Gasdermin D, Biomarkers, Surgical risk factors, Inflammatory response

## Abstract

**Background:**

Postoperative delirium (POD) is a common complication after major surgery and has been linked to perioperative neuroinflammation. Tranexamic acid (TXA), an antifibrinolytic agent, may influence inflammatory pathways, but its effect on POD remains unclear. This post hoc analysis investigated the association between intraoperative TXA administration, POD, and perioperative biomarkers of inflammation and neuronal injury.

**Methods:**

This post hoc analysis of the prospective CONFESS cohort included patients aged ≥ 60 years undergoing elective spine surgery. POD was assessed using the Nursing Delirium Screening Scale and DSM-5 criteria. Multivariable logistic regression evaluated the association between TXA and POD. Perioperative changes in IL-6, S100β, sTREM2, and Gasdermin D were analyzed using repeated-measures ANOVA. False discovery rate correction was applied for multiple testing.

**Results:**

Among 93 enrolled patients, POD screening was available for 89, of whom 42 (47.2%) received intraoperative TXA. Overall, 17 patients (18.7%) developed POD, including 12 (70.6%) who had received TXA. In univariate analysis, TXA administration was associated with POD (OR 3.36, 95% CI 1.07–10.54; *p* = 0.035), but this association was not maintained after multivariable adjustment and false discovery rate correction. Gasdermin D was the only biomarker independently associated with POD (OR = 0.212 [0.046–0.974], *p* = 0.046). Significant perioperative biomarker changes were observed, with a significant time × treatment interaction only for IL-6 (*p* = 0.028). In exploratory subgroup analyses, TXA exposure was associated with a higher incidence of POD among patients aged ≥ 75 years, whereas no association was observed in younger patients.

**Conclusion:**

In this exploratory post hoc analysis, intraoperative TXA administration was not independently associated with postoperative delirium after adjustment for relevant confounders. Although perioperative biomarker dynamics reflected the expected inflammatory response to surgery, only IL-6 demonstrated differential temporal changes according to TXA exposure, while Gasdermin D emerged as a potential biomarker associated with POD. Given the observational design, limited sample size, and small number of delirium events, these findings should be regarded as hypothesis-generating. Larger randomized studies using standardized TXA protocols are warranted to clarify the relationship between TXA, perioperative neuroinflammation, and postoperative delirium.

## Background

Postoperative delirium (POD) is a prevalent neuropsychiatric complication following major surgical interventions, particularly those of prolonged duration. It is characterized by acute alterations in consciousness, cognitive dysfunction, and disturbances in the sleep-wake cycle, typically manifesting between two and five days postoperatively [1]. Clinically, affected patients exhibit fluctuating levels of confusion, inattention, and disorganized thinking [2]. The occurrence of POD has been associated with increased requirements for intensive care, extended hospital stays, heightened medication usage, and a greater incidence of postoperative complications, ultimately contributing to elevated morbidity and mortality rates [3–5]. Furthermore, POD is implicated in long-term disability and the loss of independence, particularly in elderly patients and those with preexisting cognitive impairments [6]. POD affects approximately 10% to 70% of patients, with incidence rates being particularly elevated in older adults, individuals undergoing cardiac surgery, and those with multiple comorbidities [7]. 

POD is a frequent and serious complication in older adults undergoing major surgery, with increasing evidence implicating perioperative neuroinflammatory mechanisms in its pathogenesis. The neuroinflammatory hypothesis suggests that systemic inflammation activates microglia and astrocytes, leading to the release of pro-inflammatory cytokines such as tumor necrosis factor-α (TNF-α), as well as oxidative stress and neuronal injury markers including S100β and soluble TREM2 (sTREM2) [8]. 

Tranexamic acid (TXA), a synthetic lysine analogue with antifibrinolytic properties, is widely used in cardiac, orthopedic, and neurosurgical procedures to reduce blood loss and transfusion requirements [9]. Despite its well-established hemostatic benefits, the potential impact of TXA on neuroinflammation and POD risk remains unclear. Conflicting reports exist regarding its influence on blood–brain barrier (BBB) integrity and neurotoxicity, particularly in the context of open-heart surgery, where elevated TXA concentrations in cerebrospinal fluid have been observed [10]. Conversely, TXA-mediated reduction in intraoperative blood loss may attenuate POD risk, as substantial blood loss (> 500 mL) is a known contributor to delirium [1]. Moreover, TXA has been shown to exert immunomodulatory effects, modulating cytokine profiles and innate immune cell activation, which may further influence the neuroinflammatory cascade [11]. 

Recent exploratory studies by Hindman et al. [12, 13] suggested that TXA may be associated with a lower incidence and severity of POD with a relative risk reduction of up to 33%. Their work identified several inflammatory and neuronal injury biomarkers, including TNF-α, IL-6, IL-8, IL-10, and S100β, as potential correlates of delirium.

Based on the widely discussed neuroinflammatory hypothesis of POD pathogenesis and these exploratory findings, the present post hoc analysis investigates the association between intraoperative TXA administration and POD.

In addition, we explore perioperative changes in biomarkers of inflammation (IL-6, TNF-α), neuronal injury (S100β, sTREM2), and pyroptosis (Gasdermin D), aiming to elucidate the mechanistic pathways linking TXA to POD. We extend the biomarker panel used by Hindman et al. [12, 13] to include Gasdermin D, given its emerging role in neuroinflammatory cell death processes.

This study also explores TXA-associated differences in surgical characteristics, such as blood loss, and their potential association with POD risk. We further explore potential age-related differences in these associations, given the increased susceptibility of older patients to neuroinflammation and postoperative delirium.

This study aims to clarify TXA’s complex role as both a hemostatic and immunomodulatory agent in the context of POD, and to identify biomarker signatures that may predict susceptibility or resilience to delirium in older surgical patients.

## Methods

### Ethics, Setting and participants

The Cognitive Dysfunction following Elective Spine Surgery in Elderly Patients (CONFESS) study was designed as a prospective, single-center observational cohort study to investigate perioperative risk factors for POD and postoperative cognitive dysfunction in elderly patients undergoing elective spine surgery. The primary study hypothesis focused on the role of surgical characteristics, including duration of surgery, as risk factors for POD, alongside a broader set of predefined hypotheses addressing perioperative, inflammatory, and neurobiological mechanisms [6]. 

The present analysis of TXA represents a post hoc secondary analysis of the prospective observation cohort study CONFESS. The association between intraoperative TXA administration and POD was not a pre-specified endpoint in the original study protocol but was explored given the potential relationship between TXA use, intraoperative blood loss, and perioperative inflammatory responses, which were part of the initial conceptual framework [6]. Specifically, this analysis examines biomarkers of inflammation, oxidative stress, and neuronal damage - including IL-6, Gasdermin D, sTREM2, and S100β - to identify potential interactions contributing to POD risk.

As an observational study, CONFESS did not involve any study-specific interventions beyond routine clinical care. In routine practice at our institution, when administered, tranexamic acid (TXA) was typically given as 1 g intravenously after induction of anaesthesia and before skin incision, with an optional second 1 g dose at wound closure. No protocol-specified intraoperative re-dosing based on surgical duration was implemented. TXA administration, including its timing and dosing, was entirely at the discretion of the treating clinicians and was neither standardized nor prospectively controlled. Consequently, patients were not randomized to TXA or non-TXA groups, and treatment allocation reflects real-world clinical practice [6]. 

Patients were screened from February 2018 to March 2020 at a 950-bed tertiary care university hospital in collaboration with the Department of Anaesthesiology. Recruitment was terminated prematurely due to the SARS-CoV-2 pandemic, and data collection was completed in March 2021 [6]. 

Inclusion criteria comprised patients aged ≥ 60 years undergoing elective spine surgery without dural opening, native German speakers, and the ability to provide informed consent. Exclusion criteria included neurodegenerative or psychiatric disorders, use of CNS-active medication, inability to participate in follow-up assessments, participation in interventional studies, metallic implants, or active neoplastic disease. Chronic anticoagulant therapy was not an exclusion criterion. Patients receiving anticoagulation were included in the study and managed according to standard institutional perioperative protocols [6]. 

Of 124 initially recruited patients, 19 were excluded due to not meeting inclusion criteria or withdrawal of consent. Data from 11 patients were excluded due to incomplete datasets, and one patient was erroneously screened, resulting in a final analytical cohort of 93 patients [14]. 

### Study design and delirium assessment

Data collection was performed preoperatively, intraoperatively, postoperatively, and at a three-month follow-up in accordance with the predefined study protocol (Table 1) [14]. Preoperative assessments included a structured medical anamnesis and baseline blood sampling for immunologic, neuronal, and metabolic markers [6, 15]. 

General anesthesia was administered using a standardized sevoflurane-based regimen, and standard neurosurgical techniques were applied. Forty-four patients received TXA as an intravenous bolus (1 to 2 g, weight-dependent and surgeon dependant). Postoperatively, patients were monitored for at least six hours, followed by systematic screening for POD. Delirium assessments were conducted repeatedly during early, late, and night shifts over the first two postoperative days by trained study personnel using the Nursing Delirium Screening Scale (Nu-DESC), with positive screenings confirmed by DSM-5 criteria. If POD was detected, monitoring continued until resolution or for a maximum of seven days. Patients without POD were discharged after two days following structured chart review [14, 16, 17].

**Table 1:** Study design of CONFESS

Study period						
		Preoperative	perioperative	postoperative		
	enrolment	V0	V1	V2.1	V2.2	V3
timepoint		-2d	0d + 6h	1d	2d	3months
Neuropsychological test batterie in accordance to study protocol		x				x
Informed consent		x				
Demographic data		x				
Medical history		x				
Cognitive screening		x				
Delirium screening		x	x (at 6h)	3x	3x	
Blood samples		x	x	x	x	

Summary (Table 1): Patients were evaluated at five time points: V0, V1, V2.1, V2.2, and V3. The preoperative assessment included Neuropsychological test batterie in accordance to study protocol (Mueller et. al. 2020), informed consent clarification, collection of demographic data and medical history, cognitive screening, delirium screening, and blood sample collection. Six hours postoperatively, blood samples were drawn, and delirium screening was performed. For the following two days, delirium was assessed three times daily, and blood samples were collected each day. In the absence of postoperative delirium, patients returned for a follow-up interview three months later

Secondary outcomes were assessed using standardized and previously published questionnaires and test batteries [14]. Cognitive function was evaluated using the Consortium to Establish a Registry for Alzheimer’s Disease Plus (CERAD-Plus) test battery and the Mehrfach-Wortschatz-Intelligenztest type B (MWT-B) [18, 19]. Patient-reported quality of life was assessed using the 36-item Short Form Health Survey (SF-36) and the Patient-Reported Outcomes Measurement Information System 29-item profile (PROMIS-29) [20, 21]. Frailty was measured with the Groningen Frailty Indicator, functional disability with the Oswestry Disability Index, and symptoms of anxiety and depression with the Hospital Anxiety and Depression Scale (HADS) [22]. 

### **Perioperative surgical and anaesthesiologic procedures**

Surgical and anesthetic procedures followed national guidelines and in-house protocols without modification for the study. Patients were positioned prone except for ventral fusion cases, which required a supine position. Standard surgical equipment included an operating microscope and a mobile X-ray device. Procedures were categorized by spine levels, complexity (simple, intermediate, complex), and type of intervention (cervical, thoracic, lumbar decompression and/or fusion, kyphoplasty).

Anesthesia was induced with Sufentanil (0.3–0.6 µg/kg), Propofol (1.5–2.5 mg/kg), and Cisatracurium (0.15 mg/kg) for muscle relaxation. Maintenance involved Sevoflurane at 0.8–1.0x MAC. Thirteen patients received morphine-derived opioids intraoperatively or in the immediate postoperative period as part of standard anesthetic analgesia, whereas 19 patients had documented chronic preoperative opioid use. Anesthetic depth was continuously monitored via bispectral index EEG. Fluid loss was replaced with isotonic electrolyte solution, and hypotension was managed with fluids and norepinephrine as needed. Vital parameters – including SpO₂, ECG, and BP - were tracked from 30 min before induction, with BP measured every five minutes or via arterial catheter when necessary [14]. 

### Measurement of Biomarkers

Routine biomarkers (leukocyte count and C-reactive protein) were measured as part of routine clinical care, while study-specific markers were preserved and compared between POD-positive and POD-negative cohorts, matched by age and sex. Biosamples were analyzed at multiple time points (V0, V1, V2.1, V2.2).

From V0 to V2, the sera for biomarker analysis were sampled. Until analysis, they were stored at -80 °C and pseudonymized according to subject and draw time. Sera will be stored for 10 years [14]. 

Enzyme-linked immunosorbent assays (ELISA) using the sandwich ELISA principle were executed via the ELISA reader Infinite M200 Pro, TECAN Group Ltd, Switzerland. The manufacturer’s instructions we followed (Human IL-6 Uncoated ELISA; dataHuman TREM2 ELISA Kit; Gasdermin D ELISA, Horizon Bioscience Discovery Ltd; S100 ELISA cobas e 411, Roche). Photometric absorbance measurement was executed at a 450 nm wavelength by the appropriate microplate reader (ELISA reader). Standard curve generation and concentration calculations were performed through the statistical software GraphPad PRISM 8.0.1. CRP values were gauged in the central laboratory facility of the University Medicine Greifswald (using Dimension Vista, Siemens Healthcare Diagnostics, Eschborn, Germany) [15]. 

### Data management, sample size considerations, and statistics

Study data were initially recorded on paper and transferred to an electronic case report form (CentraXX, Kairos GmbH, Bochum, Germany). Participants were pseudonymized using numerical codes; identifying data were stored separately. Study data and questionnaires were entered directly into the digital system. Paper records will be archived for ten years, with digital data retained for analysis within the study team and clinical network. Statistical analyses were performed using SPSS (version 28, IBM, Armonk, USA). Descriptive statistics are presented as means ± standard deviation for continuous variables and as absolute and relative frequencies for categorical variables. Inferential analyses included p-values, odds ratios (OR), and unstandardized beta coefficients with 95% confidence intervals (95% CI), where applicable.

All secondary and exploratory analyses were considered hypothesis-generating.

To account for multiple comparisons, the Benjamini–Hochberg false discovery rate (FDR) procedure was applied separately for predefined families of analyses. First, FDR correction was performed across the four multivariable logistic regression models evaluating the association between intraoperative TXA administration and postoperative delirium. Second, FDR correction was applied to the four investigated biomarkers (IL-6, Gasdermin D, S100β, and sTREM2). A threshold of q < 0.05 was considered statistically significant.

No a priori sample size calculation was performed for the present post hoc analysis of TXA-related outcomes. The available cohort was determined by the CONFESS study recruitment period and eligibility criteria.

The occurrence of POD was examined in all subjects from V0 to V2 and was then compared between subjects who received intraoperative TXA and those who did not. The association between TXA administration, POD, and clinical variables was assessed using multivariable binary logistic regression.

Due to the relatively small number of POD events in the cohort, a stepwise regression modelling approach was applied. In situations with a limited number of events, inclusion of a large number of covariates in a single multivariable model may lead to overfitting, unstable estimates, and a low events-per-variable ratio. To mitigate these risks, variables were introduced in sequential blocks across Models 1 to 4. Covariates were selected apriori based on biological plausibility, previous literature on established risk factors for postoperative delirium, and the predefined perioperative variables available within the CONFESS study, rather than by automated statistical selection procedures. This modelling strategy was intended to provide a transparent assessment of the association between TXA and POD while progressively accounting for potential confounding.

Four models were constructed: Model 1 examined TXA administration alone; Model 2 adjusted for age and surgical duration; Model 3 incorporated biomarker differences (ΔsTREM2, ΔIL-6, ΔGasderminD, and ΔS100β); Model 4 included additional perioperative factors (BMI, fluid intake, fluid output, fluid balance, and intraoperative blood loss). Subgroup analyses were conducted to evaluate the interaction between TXA and patient age. All statistical tests were two-tailed, with significance set at *p* < 0.05.

A detailed description of all covariates included in each regression model is provided in Table 2.

**Table 2:** Description of all covariates

Covariate	Definition	Type/Unit	Included in Models
TXA administration	Intraoperative administration of tranexamic acid (yes/no) according to routine clinical practice	Binary	Models 1-4
Age	Age at surgery	Continuous (years)	Models 2-4
Duration of surgery	Time from skin incision to wound closure	Continuous (minutes)	Models 2-4
ΔIL-6	Difference between postoperative (V1) and preoperative (V0) serum IL-6 concentration	Continuous (pg/mL)	Model 3
ΔGasdermin D	Difference between V1 and V0 serum Gasdermin D concentration	Continuous (ng/ml)	Model 3
ΔsTREM2	Difference between V1 and V0 serum soluble TREM2 concentration	Continuous (ng/ml)	Model 3
ΔS100β	Difference between V1 and V0 serum S100β concentration	Continuous (pg/ml)	Model 3
BMI	Body mass index	Continuous (kg/m²)	Model 4
Fluid intake	Total intraoperative intravenous fluid administration	Continuous (ml)	Model 4
Fluid output	Total intraoperative fluid loss (urine and blood loss)	Continuous (ml)	Model 4
Fluid balance	Fluid intake minus fluid output	Continuous (ml)	Model 4
Intraoperative blood loss	Estimated blood loss during surgery	Continuous (ml)	Model 4

Summary (Table 2): Definitions of all covariates included in the multivariable regression models

To also assess changes in biomarker levels over time and between groups, a repeated measures analysis of variance (ANOVA) was conducted with time (V0 vs. V1) as a within-subject factor and TXA exposure (given/not given) as a between-subject factor.

The primary effect of interest was the time × treatment interaction, which tests whether the change in biomarker levels over time differs between the TXA and control groups, corresponding to a difference-in-differences approach.

TXA was evaluated as an exposure variable for postoperative delirium rather than as a hemostatic intervention; therefore, no sample size calculation was performed for TXA-related effects.

## Results

### Study population and data availability

Among 93 enrolled subjects, postoperative delirium (POD) screening data were available for 89 patients. Of the 89 patients with available POD screening, 42 (47.19%) received TXA and 47 (52.81%) did not.

**Fig. 1:**
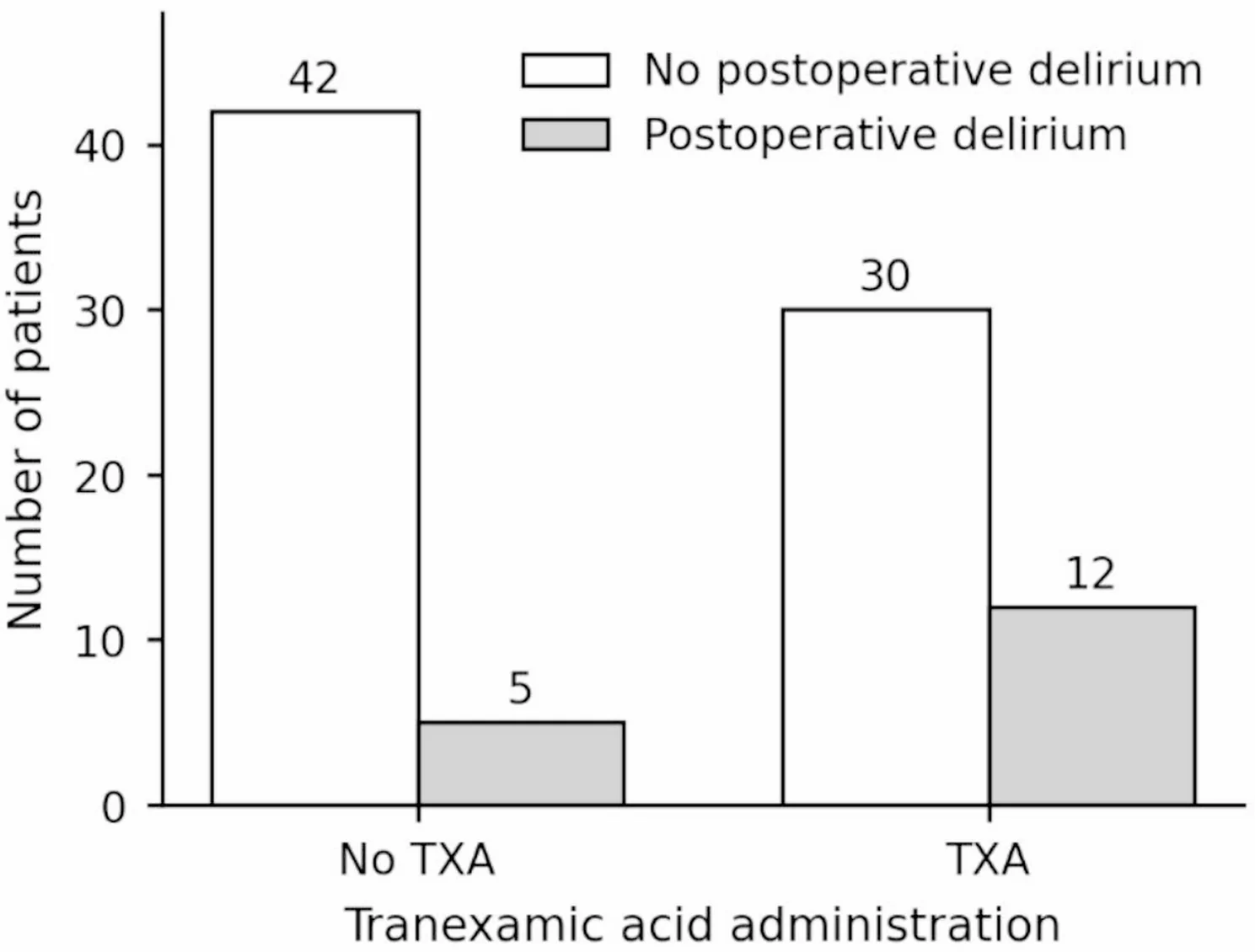
The occurrence of postoperative delirium in relation to the application of tranexamic acid Summary (Figure 1): The graph illustrates the incidence of postoperative delirium among patients, categorized by the administration of tranexamic acid (TXA) during surgery. The first two bars depict the number of patients who did or did not develop delirium without TXA administration. The second two bars represent the number of patients who did or did not develop delirium following TXA administration during surgery

17 (18.7%) of the 89 patients with POD screening developed POD, while 72 (81.3%) did not. Among the 17 patients who developed POD, 12 (70.59%) had received TXA intraoperatively. TXA administration was significantly associated with POD development (OR = 3.36 [1.07–10.54], *p* = 0.035). A graphical overview of these results is presented in Fig. 1; Table 3.

**Table 3:** Chi-square test within a cross-table

	Tranexamic acid		
Postoperative delirium	no	yes	line total
No	42	30	72
Yes	5	12	17
Column total	47*	42*	n=93

Summary (Table 3): This chart shows the distribution of patients with or without tranexamic acid (TXA) regarding postoperative delirium. Among 72 patients without postoperative delirium, 30 received TXA. Among 17 patients with postoperative delirium, 12 received TXA. In total, 47 patients did not receive TXA, 5 of whom experienced postoperative delirium. Of the 42 patients who received TXA, 12 experienced postoperative delirium. *+ 2 missing POD screening

Regarding surgical experience, 70 (75.27%) patients were operated on by an experienced senior physician and 23 (24.73%) by other certified neurosurgeons. In the TXA group (including all enrolled patients, not restricted to those with available POD screening assessments), 36 of the 44 (81.82%) patients were treated by the senior physician and 8 (18.18%) by other neurosurgeons. In the non-TXA group, the senior physician operated 34 of 49 (69.39%) patients on. (TXA vs. non-TXA: *p* = 0.17, OR = 1.99 [0.74–5.35]).

A detailed overview of baseline sociodemographic and surgical characteristics for the overall cohort, as well as stratified by TXA exposure, is provided in Table 4.1. POD severity, assessed using CAM-S and Nu-DESC scores, is summarized in Table 4.2.

**Table 4:** A Patients characteristics with numbers and percentages of certain attributes. B Outcome comparison in relation to the development of POD

A					
	total	With TXA	Without TXA	p-value With TXA vs. Without TXA	*Drop-outs or missing (n=31)*
Sex Male Female	45 48	22 22	23 26	-	*16* *15*
Age	71.28±6.79 (n=93)	70.48±6.98 (n=44)	72±6.6 (n=49)	p=0.319	*74.26±8.37*
BMI	29.05±4.71 (n=91)	29.53±5.7 (n=43)	28.62±3.61 (n=48)	p=0.7615	*30.20±7.40 (n=5)*
Years of education	9.43±1.484 (n=93)	9.522±1.501 (n=44)	9.346±1.479 (n=49)	p=0.5907	*No data*
Education type (in school years) 0 1-10 12-13	(n=93) 0 79 14	(n=44) 0 37 7	(n= 49) 0 42 7	-	*No data*
Operating physician (%) Experienced senior physician Other certified neurosurgeons	(n=93) 70 (75.27%) 23 (24.73%)	(n=44) 36 (81.83%) 8 (18.18%)	(n= 49) 34 (69.39%) 15 (30.61%)	p=0.17	*(n = 6)* *4 (66.66%)* *2 (33.33%)*
Duration of surgery (in minutes)	187.267±106.757 (n=86)	231.4±105.614 (n=40)	148.89±92.86 (n=46)	**p<0.0001**	*241.44±136.19 (n=9)*
Spinal levels	2.6145±1.471 (n=83)	2.945±1.489 (n=37)	2.347±1.417 (n=46)	**p=0.0146**	*2.86±1.35 (n=7)*
Fluid in (in milliliters)	2469.27±1290.32 (n=83)	2913.15±1263.65 (n=38)	2094.44±1106.91 (n=45)	**p=0.0012**	*3714.29±1621.5 (n=7)*
Fluid out (in millilitres)	218.05±369.64 (n=59)	298.7±439.8 (n=17)	150±288.03 (n=32)	p=0.1142	*344.60±287.00 (n=5)*
Fluid balance (in millilitres)	2359.12±1056.38 (n=86)	2707.79±1070.78 (n=43)	2010.46±928.95 (n=43)	**p=0.0003**	*3213.83±1756.20 (n=6)*
Intraoperative blood loss (in millilitres)	270.80±442.50 (n=87)	416.13±530.85 (n=44)	122.09±260.1 (n=43)	**p=0.0016**	*633.33±469.04 (n=6)*
IL-6 in pg/ml V0 V1 p-value	6.75±26.56 (n=38) 13.98±28.83 (n=38) **p<0.0001**	1.96±2.14 (n=18) 14.87±19.76 (n=18) **p<0.0001**	11.07±36.45 (n=20) 13.19±35.61 (n=20) p=0.2287	p=0.3647 **p=0.0240**	*No data*
GasderminD in ng/ml V0 V1 p-value	1.61±1.05 (n=37) 2.07±1.16 (n=37) p=0.0536	1.65±1.3 (n=18) 2.17±1.26 (n=18) p=0.2231	1.56±0.79 (n=19) 1.98±1.1 (n=19) p=0.249	p=0.5628 p=0.4563	*No data*
sTREM2 in ng/ml V0 V1 p-value	106.67±58.2 (n=38) 90.91±42.73 (n=37) p=0.3463	110.61±69.35 (n=18) 82.39±46.03 (n=18) p=0.3759	103.13±47.6 (n=20) 98.98±38.84 (n=19) p=0.7677	p=0.6984 p=0.1571	*No data*
S100β in pg/ml V0 V1 p-value	0.06±0.03 (n=36) 0.67±0.65 (n=37) **p<0.0001**	0.05±0.03 (n=17) 0.66±0.36 (n=17) **p<0.0001**	0.06±0.03 (n=19) 0.68±0.83 (n=20) **p<0.0001**	p=0.6525 p=0.2658	*No data*

B					
	total	With TXA	Without TXA	p-value With TXA v.s Without TXA	
Number of patients With POD Without POD	93^+^ 17 (18.3%) 72 (77.4%)	44* 12 (27.3%) 30 (68.2%)	49“ 5 (10.2%) 42 (85.7%)	POD: p=0.0566	
Delirium strength CAM-S	9±3.785 (n=7, 7.5%)	9.833±3.371 (n=6, 13.6%)	4 (n=1, 2.0%)	n too small	
Delirium strength Nu-DESC	2.44±2.27 (n=25, 26.9%))	2.875±2.70 (n=16, 36.4%)	1.66±0.86 (n=9, 18.4%)	p=0.5214	

Summary (A): patient characteristics with 93 data sets. Patients were characterized according to sex; age; BMI; years of education; education type; operating physician; duration of surgery in minutes; spinal levels affected during surgery; instilled fluid; lost fluid; fluid balance; intraoperative blood loss; biomarker levels of IL-6, GasderminD, sTREM2, S100β at V0 and V1. Education type was differentiated into no education, ability to absolve an apprenticeship (1-10 school years - secondary school diploma or intermediate high school), and ability to attend university (12 to 13 school years - graduation from high school, technical college entrance qualification). P-values between the TXA and the non-TXA group were calculated.Addition: patients characteristics of drop-outs or missing, if data acquisition was possible

Summary (B): patient characteristics with 93 data sets. Patients were characterized according to their development of POD; delirium strength measured through the CAM-S-score; delirium strength measured through the Nu-DESC-score. P-values between the TXA and the non-TXA group were calculated. *42 with POD screening; “47 with POD screening ^+^89 with POD screening

### Biomarker analysis

Out of 93 patients, sera from 38 (IL-6, sTREM2 at V0; 40.86%), 37 (sTREM2 at V1, Gasdermin D, S100 at V1; 39.78%), and 36 (S100 at V0; 38.71%) were examined.

Biomarker levels of IL-6, Gasdermin D, and S100β increased from V0 to V1, whereas sTREM2 showed a slight decrease.

In the overall cohort, paired analyses demonstrated significant perioperative changes between V0 and V1 for IL-6 (*p* < 0.0001) and S100β (*p* < 0.0001), whereas no significant changes were observed for Gasdermin D (*p* = 0.0536) or sTREM2 (*p* = 0.3463), see Table 2.

The same analyses were then performed separately for the TXA and non-TXA groups. In the TXA group, significant changes between V0 and V1 were observed for IL-6 (*p* < 0.0001) and S100β (*p* < 0.0001), while Gasdermin D (*p* = 0.2231) and sTREM2 (*p* = 0.3759) did not change significantly.

In the non-TXA group, only S100β showed a significant perioperative increase (*p* < 0.0001), whereas IL-6 (*p* = 0.2287), Gasdermin D (*p* = 0.2490), and sTREM2 (*p* = 0.7677) remained unchanged.

When biomarker levels were compared between the TXA and non-TXA groups, a significant between-group difference was observed only for IL-6 at V1 (*p* = 0.0240).

Perioperative biomarker levels showed significant time-dependent changes. After correction for multiple testing using the Benjamini–Hochberg FDR procedure across four biomarkers, significant associations were observed for S100β (*p* < 0.001, q = 0.004), IL-6 (*p* = 0.003, q = 0.006), Gasdermin D (*p* = 0.004, q = 0.0067), and sTREM2 (*p* = 0.023, q = 0.031), indicating robust perioperative biomarker dynamics.

### Multivariable analysis of factors associated with pOD (Table 5, Models 1 to 4)

A multiple binary regression analysis was conducted to assess the association between TXA, POD, and other influencing factors. The Benjamini-Hochberg false discovery rate (FDR) was applied across the four regression models to adjust for multiple testing.

**Table 5:** Regression chart for TXA

		Model 1 (tranexamic acid application)		Model 2 (tranexamic acid application, age, duration of surgery)		Model 3 (tranexamic acid application, age, duration of surgery, mean difference of biomarker levels between V0 and V1)		Model 4 (tranexamic acid application, age, duration of surgery, mean difference of biomarker levels between V0 and V1, BMI, fluid intake, fluid outtake, fluid balance, intraoperative blood loss)	
		Odds-Ratio	p-value	Odds Ratio	p-value	Odds Ratio	p-value	Odds Ratio	p-value
POD	TXA in g	5.851 (1.374-24.925)	**0.017**	7.39 (1.144-47.745)	**0.036**	9.196 (0.928-91.141)	0.58	0.00 (0.00-0.00)	0.999
	Age	-	-	1.166 (0.959-1.418)	0.123	1.215 (0.980-1.507)	0.076	24.072 (0.00-0.00)	0.998
	Duration	-	-	1.007 (0.998 – 1.015)	0.114	1.008 (0.996 – 1.019)	0.203	1.133 (0.00 – 8.353E+187)	1.0
	BMI							0.00 (0.00-0.00)	0.999
	Fluid intake							0.973 (0.00-2.269E+11	0.998
	Fluid outtake							1.101 (0.00-1.21E+40)	0.998
	Fluid balance								
	Intraoperative blood loss							1.090 (0.00-2.33E+45)	0.999
	ΔsTREM2	-	-	-	-	0.99 (0.97-1.011)	0.341	0.625 (0.00-1.327E+170)	0.998
	ΔIL-6	-	-	-	-	1.055 (0.883 – 1.26)	0.555	10.727 (0.00-0.00)	0.999
	ΔGasderminD	-	-	-	-	0.212 (0.046-0.974)	**0.046**	0.00 (0.00-0.00)	0.997
	ΔS100β	-	-	-	-	1.63 (0.306 – 8.677)	0.567	0.00 (0.00-0.00)	0.998

Summary (Table 5): Regression chart for tranexamic acid application concerning postoperative delirium (Model 1). Correlated with age of patients and duration of surgery in a separate block in Model 2. Correlated with biomarkers (ΔsTREM2, ΔIL-6, ΔGasderminD, ΔS100β) in a separate block in Model 3. Correlated with BMI, fluid intake, fluid outtake, fluid balance, intraoperative blood loss in the same block as age and duration of surgery in Model 4. Within the odds ratio, the lowest and highest numbers were given at a 95% confidence interval

TXA administration in Model 1 was associated with an increased odds of POD (*p* = 0.017, OR = 5.851 [1.374–24.925]) but did not remain significantly associated after adjustment (q = 0.068).

In Model 2, a multivariable model adjusting for age and duration of surgery, TXA remained significant (*p* = 0.036, OR = 7.39 [1.144–47.745]), while age (*p* = 0.123, OR = 1.166 [0.959–1.418]) and surgery duration (*p* = 0.114, OR = 1.007 [0.998–1.015]) were not significant. After applying for multiple testing correction, this association of TXA did not remain significant (q = 0.072).

Model 3 incorporated all biomarkers, age, duration of surgery, and TXA. In this model, only Gasdermin D was significantly associated with POD (*p* = 0.046, OR = 0.212 [0.046–0.974]); all other variables were not significant (FDR [TXA]: q = 0.077).

In Model 4, additional clinical variables (BMI, fluid intake, fluid output, fluid balance, and intraoperative blood loss) were included alongside the clinical parameters age and surgery duration. None of these variables showed a statistically significant association with the development of POD (FDR [TXA]: q = 0.238).

### Time × Treatment Interaction Analysis

Repeated measures ANOVA demonstrated significant time-dependent changes for all investigated biomarkers, including IL-6 (*p* = 0.003, η²*p* = 0.220), Gasdermin D (*p* = 0.004, η²*p* = 0.218), sTREM2 (*p* = 0.023, η²*p* = 0.140), and S100β (*p* < 0.001, η²*p* = 0.467), indicating moderate to large perioperative effects. The repeated-measures ANOVA showed no significant statistical main effects of TXA exposure for any biomarker (all *p* ≥ 0.674, η²*p* ≤ 0.005).

A significant time × treatment interaction was observed only for IL-6 (*p* = 0.028, η²*p* = 0.127), indicating differential changes over time between TXA and non-TXA groups. In contrast, no significant interactions were found for Gasdermin D (*p* = 0.736, η²*p* = 0.003), sTREM2 (*p* = 0.181, η²*p* = 0.051), or S100β (*p* = 0.990, η²*p* < 0.001).

A significant time × treatment interaction was observed for IL-6 (*p* = 0.028), which remained significant after FDR correction (q = 0.037), suggesting differential IL-6 trajectories between TXA and non-TXA groups.

### Age-Stratified and Adjusted Analyses

To assess age-dependent effects of TXA application on POD, 89 subjects with complete POD screening were stratified into ≥ 75 years and < 75 years.

In patients ≥ 75 years (*n* = 37), POD occurred in 10 of 17 patients receiving TXA (58.82%) compared with 3 of 20 patients without TXA (15.00%), corresponding to a significantly association of TXA with POD in the age group ≥ 75 years (OR = 8.10 [1.70–38.57], *p* = 0.0075).

In patients < 75 years (*n* = 52), POD occurred in 2 of 25 patients receiving TXA (8.00%) compared with 2 of 27 patients without TXA (7.41%). This difference was not statistically significant (OR = 1.09 [0.14–8.46], *p* = 1.000).

## Discussion

### Principal Findings

In this study, we did not observe a protective effect of intraoperative TXA administration on the incidence of POD. Unadjusted univariate analyses suggested higher POD rates among patients receiving TXA (OR = 5.851, *p* = 0.017). This association did not persist after adjustment for confounding variables and correction for multiple testing. This pattern highlights the importance of accounting for perioperative and demographic factors when evaluating associations between TXA exposure and postoperative neurocognitive outcomes, and suggests that crude associations may reflect underlying differences in patient and procedural characteristics.

### Multivariable Analysis and Confounding

In the fully adjusted regression model, none of the investigated perioperative variables (including BMI, fluid administration and balance, intraoperative blood loss, age, or duration of surgery) showed a statistically significant association with POD. This likely reflects both limited statistical power due to the relatively small number of POD events and the increased model complexity introduced by multiple covariates.

TXA remained significantly associated with POD after adjustment for age and surgical duration (Model 2), but this association was attenuated in more comprehensively adjusted models. This pattern indicates that the observed relationship is sensitive to model specification and underscores the importance of robust multivariable adjustment when interpreting observational associations. Although covariates were introduced in sequential models to reduce model complexity, some degree of model overfitting cannot be excluded.

Given the small cohort and limited number of POD events, all adjusted analyses were exploratory. Although TXA was associated with POD in univariate analysis and after limited adjustment, this association did not remain significant after multiple-testing correction and was not robust in more comprehensively adjusted models. Furthermore, missing data, collinearity, low event-per-variable ratios, and incomplete model convergence limited the multivariable analyses. As an observational study with non-randomized TXA administration, residual confounding cannot be excluded, and heterogeneity in surgical procedures may have further influenced the results. Accordingly, these findings should be interpreted with caution and confirmed in larger, prospective cohorts.

### Biomarker Differences

Among the investigated biomarkers, only Gasdermin D showed a statistically significant association with POD, supporting a potential role in pyroptosis-related neuroinflammatory pathways [23]. In contrast, TXA exposure was not consistently associated with perioperative biomarker changes. Although all biomarkers changed significantly over time from V0 to V1, reflecting the expected physiological response to surgery, only IL-6 demonstrated a significant time × treatment interaction. No interaction was observed for Gasdermin D, sTREM2, or S100β. These findings indicate that TXA was not associated with consistent alterations in perioperative inflammatory or neuronal injury trajectories. The isolated IL-6 interaction should therefore be interpreted cautiously and warrants further mechanistic investigation. As treatment effects are best assessed by interaction analyses rather than within-group comparisons, the absence of consistent interaction effects across biomarkers argues against a robust effect of TXA on perioperative biomarker dynamics.

IL-6 was significantly elevated postoperatively in the TXA group, although this appeared primarily driven by baseline differences and did not translate into an independent association with POD. Surgical duration was not significantly associated with POD, despite previous reports [6, 7].

Given the exploratory nature of this post hoc analysis and the limited sample size, these biomarker findings should be interpreted as hypothesis-generating and require confirmation in larger prospective studies.

### Comparison with Previous Studies

Our findings do not replicate the protective association reported by Hindman et al. [12, 13], who demonstrated a reduction in POD incidence and severity in a randomized placebo-controlled trial of standardized spinal fusion procedures. Their protocol employed fixed dosing regimens (10 mg/kg bolus followed by 2 mg/kg/h infusion of TXA) and reported associations between TXA exposure and inflammatory biomarkers, including IL-6, IL-10, and S100β, as well as reduced TNF-α levels. Elevated postoperative S100β levels were associated with reduced delirium severity in the TXA group, suggesting a potential neuroprotective effect.

In contrast, we did not observe consistent TXA-related effects on biomarker trajectories. Aside from the observed IL-6 interaction signal and a significant time × treatment interaction for IL-6, no robust or reproducible associations were identified between TXA exposure and inflammatory or neuronal injury markers.

Several key differences may explain these discrepancies, including the more heterogeneous surgical population in our study, higher patient age (mean age 71 vs. 59 years), and the absence of a standardized TXA dosing protocol.

Notably, Gasdermin D, an emerging marker of pyroptosis-related neuroinflammation [23], was included in our analysis and showed an association with POD. This pathway was not assessed in prior TXA studies and may represent a previously unexplored mechanism relevant to perioperative neuroinflammatory responses.

### Methodological Considerations and Limitations

Several limitations should be considered. TXA dosing was relatively conservative compared with orthopedic and neurosurgical protocols using doses up to 100 mg/kg without a clear increase in adverse events [9, 24]. Lower doses may reduce adverse effects such as seizures but also limit potential anti-inflammatory or neuroprotective effects. In addition, TXA administration was not standardized, no re-dosing protocol was applied, and serum concentrations were not measured, potentially resulting in heterogeneous exposure.

Patients receiving chronic anticoagulation were included, increasing external validity but also heterogeneity. As TXA was not randomly assigned, residual confounding and selection bias, including surgeon-specific prescribing preferences, cannot be excluded. Multicenter randomized studies are therefore needed.

Exploratory analyses suggested a stronger association between TXA and POD in patients aged ≥ 75 years, consistent with the greater neurocognitive vulnerability of older adults [25], but these findings require confirmation. Additional limitations include incomplete adjustment for perioperative benzodiazepine and opioid exposure, established risk factors for POD [1], and limited generalizability due to the exclusion of patients with neurodegenerative or psychiatric disorders.

Finally, attrition between recruitment and analysis (124 enrolled, 31 excluded; Table 2.1) may have introduced selection bias. Although comparisons were limited by incomplete data, excluded patients appeared older and to have undergone more complex procedures, suggesting that higher-risk individuals may be underrepresented in the final analytic cohort.

## Conclusion

In this post hoc analysis of a prospective observational cohort, intraoperative TXA administration was not independently associated with postoperative delirium after adjustment for relevant clinical and perioperative factors.

In contrast to previous reports like those by Hindman et al. [12, 13], we found no evidence of a consistent association between TXA exposure and perioperative biomarker trajectories. Although all investigated biomarkers showed significant time-dependent perioperative changes, only IL-6 demonstrated a differential trajectory between TXA and non-TXA groups, while Gasdermin D was independently associated with POD. Given the observational design, limited sample size, and small number of POD events, these biomarker findings should be interpreted as hypothesis-generating.

Future adequately powered randomized studies using standardized TXA protocols, together with age-stratified analyses and integration of pharmacokinetic and pharmacogenetic data are needed to further clarify the relationship between TXA, perioperative neuroinflammation, and postoperative delirium.

## Data Availability

The data that support the findings of this study are available from the corresponding author, RF, upon reasonable request.

## Declarations

Subjects have given their written informed consent or in case of mental incapacity their legal representatives.
